# 1659. Prokinetic Antibiotic Agents in the Neonatal Intensive Care Unit: Ripe for Antibiotic Stewardship Efforts!

**DOI:** 10.1093/ofid/ofad500.1492

**Published:** 2023-11-27

**Authors:** Jacqueline Magers, Pavel Prusakov, Michael Storey, Pablo J J Sanchez

**Affiliations:** Nationwide Children's Hospital, Columbus, Ohio; Nationwide Children's Hospital, Columbus, Ohio; Nationwide Children's Hospital, Columbus, Ohio; Nationwide Children's Hospital, The Ohio State University College of Medicine, Columbus, Ohio

## Abstract

**Background:**

Intolerance to enteral nutrition among infants in the neonatal intensive care unit (NICU) may lead to the use of prokinetic agents to improve gastric emptying and feeding tolerance. Two such oral prokinetic agents are erythromycin and amoxicillin-clavulanate that have been used in cases of dysmotility due to intestinal failure. However, dosing strategies vary and the recommended duration of therapy has not been determined. Moreover, the use of these oral antibiotics in the NICU may fall under the radar of neonatal antimicrobial stewardship programs. Our objective was to quantify their use in the Level 4 outborn NICU at Nationwide Children’s Hospital, Columbus, OH in order to inform antibiotic stewardship efforts.

**Methods:**

This was a retrospective cohort study of all infants who received enteral erythromycin or amoxicillin-clavulanate in the NICU. The number of doses of each antibiotic was obtained from the Nationwide Children’s Hospital Pharmacy database. Pertinent clinical, laboratory, and outcome data were obtained.

**Results:**

From January 1, 2022 to April 30, 2023, 15 infants in the NICU received ≥1 dose of erythromycin ethylsuccinate and 19 infants received ≥1 dose of amoxicillin-clavulanate enterally. Erythromycin was always used to improve motility while amoxicillin-clavulanate served as a prokinetic agent in 53% of infants in whom it was prescribed. Demographics and reason for initiation are provided in Table 1. The median (IQR) doses per patient for erythromycin was 64 (28, 125; dosing interval varied from every 6 to every 8 hours) and for amoxicillin-clavulanate was 50 (9, 105) doses per patient (majority of doses were given on a BID schedule).

Characteristics of infants who received prokinetic agents in the NICU

**Figure:**
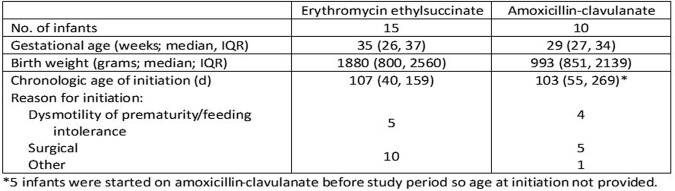

**Conclusion:**

Enteral erythromycin and amoxicillin-clavulanate have not been the target of antimicrobial stewardship programs. These antibiotics may contribute to dysbiosis and adverse events in NICU infants. An automatic stop time with a time-out when ordered in the electronic health record may be a viable strategy to reduce overutilization of these antibiotics.

**Disclosures:**

**Pavel Prusakov, PharmD**, Merck & Co: Grant/Research Support|Merck & Co: Employee **Michael Storey, PharmD, MS**, CSL Behring: Advisor/Consultant|Sarepta Therapeutics: Advisor/Consultant

